# Dissolution and antioxidant potential of apigenin self nanoemulsifying drug delivery system (SNEDDS) for oral delivery

**DOI:** 10.1038/s41598-024-59617-z

**Published:** 2024-04-17

**Authors:** Boontida Morakul, Veerawat Teeranachaideekul, Waree Limwikrant, Varaporn Buraphacheep Junyaprasert

**Affiliations:** 1 Department of Pharmacy, Faculty of Pharmacy, Mahidol University, 447 Sri-Ayuthaya Road, Rajathevi, Bangkok, 10400 Thailand; 2 Department of Manufacturing Pharmacy, Faculty of Pharmacy, Mahidol University, Bangkok, 10400 Thailand

**Keywords:** Drug delivery, Food nanotechnology

## Abstract

Self-nanoemulsifying drug delivery systems (SNEDDS) have been used to improve the oral bioavailability of various drugs. In the current study, apigenin was developed as SNEDDS to solve its dissolution problem and enhance oral bioavailability and antioxidant potential. SNEDDS were prepared by mixing Gelucire 44/14, Tween 80, and PEG 400 under controlled conditions. The droplet of diluted SNEDDS demonstrated a spherical shape with a size of less than 100 nm and a neutral charge. The very fast self-emulsification was obtained within 32 s, and the transmittance values exceeded 99%. The highest drug loading was 90.10 ± 0.24% of the initial load with the highest %encapsulation efficiency of 84.20 ± 0.03%. FT-IR and DSC spectra showed no interaction between components. The dissolution in buffer pH 1.2, 4.5, and 6.8 showed significantly higher dissolved apigenin than the apigenin coarse powder. The dissolution profiles were fitted to the Korsmeyer–Peppas kinetics. The cellular antioxidant activities in Caco-2 cells were approximately 52.25–54.64% compared to no treatment and were higher than the apigenin coarse powder (12.70%). Our work highlights the potential of SNEDDS to enhance the dissolution and permeability of apigenin and promote antioxidant efficacy, which has a strong chance of being developed as a bioactive compound for nutraceuticals.

## Introduction

Apigenin is a major plant flavone derived from the Apium genus in the family Apiaceae, also known as the celery, carrot, or parsley family^1^. A great number of studies have revealed that apigenin is a bioactive compound that has a wide range of interesting pharmacological activities and nutraceutical potential, especially the well-known antioxidant properties^2,3^. Moreover, it has anti-inflammatory, anti-autoimmune, antihyperglycemic, antineurodegenerative, and anticancer activities^4–6^. Apigenin showed the high antioxidant capacity in 2,2-diphenyl-1-picrylhydrazyl (DPPH) and 2,2-azinobis-(3-ethylbenzothiazoline-6-sulfonate (ABTS^+^) colorimetric assays and photoprotective activity against UVA and UVB radiation in human keratinocytes^7,8^. Jung^9^ found that apigenin could reduce hydrogen peroxide (H_2_O_2_)-induced oxidative damage and cellular dysfunction in the mouse osteoblastic cell line MC3T3-E1. Al-Rawi et al.^10^ reported the antioxidant efficacy of apigenin by finding a significant decrease in brain tissue peroxidation in H_2_O_2_-induced oxidative stress in adult male rats. Due to these benefits, apigenin has gained attention for use in supplements and nutraceuticals. With a molecular weight of 270.24 Da, apigenin is soluble in dimethyl sulfoxide and hot ethanol but insoluble in water which is classified as a Biopharmaceutical classification system (BCS) Class II^11^. Therefore, its absorption and oral bioavailability are limited due to its poor water solubility.

The limited water solubility of active compounds is the major obstacle in the development of delivery systems. To obtain good bioavailability upon oral administration, the active compound has to be able to dissolve in the gastrointestinal (GI) fluid before its absorption into the systemic circulation^12^. Various delivery systems, including lipid-based delivery systems, have been employed to improve the dissolution and bioavailability of poorly water-soluble compounds. SNEDDS are one type of lipid-based delivery. They are anhydrous homogeneous liquid mixtures, composed of oil, surfactant, cosurfactant, and active compounds that spontaneously form transparent nanoemulsions upon aqueous dilution with the gentle agitation of GI motility^13^. SNEDDS provide advantages over ready-to-use nanoemulsions including improvement in the stability, the possibility of being manufactured as unit dosage forms, and an increase in patient acceptability. The components of SNEDDS help increase drug loading, dissolution, and permeability, which leads to an improvement in oral bioavailability^14^. In this study, a bioactive compound, apigenin, was loaded into SNEDDS to overcome its dissolution problem. The aim of the present work was to formulate, optimize, and characterize apigenin-loaded SNEDDS, as well as evaluate the dissolution and preliminary antioxidant potentials of the optimized formulations in Caco-2 cells to obtain good biopharmaceutical attributes for administration as oral supplements.

## Materials and methods

### Materials

Apigenin was purchased from Xian Neo Biotech, Xian, China. Corn oil was purchased from Morakot Industries, Thailand, and sunflower oil was obtained from Lam Soon, Thailand. Gelucire 44/14 (lauroyl polyoxyl-32 glycerides) and Transcutol HP (diethylene glycol monoethyl ether) were kindly supplied by Gattefossé, France. Myritol 318 (caprylic/capric triglyceride) was purchased from Chemipan, Thailand. Tween 20 (polyoxyethylene sorbitan monolaurate) and Tween 80 (polyoxyethylene sorbitan monooleate) were obtained from Ajax Finechem, Australia. Span 20 (sorbitan laurate) was obtained from Croda, Singapore. Span 80 (sorbitan monooleate) and Cremophor-RH40 (PEG-40 hydrogenated castor oil) were purchased from Namsiang, Thailand. PEG 400 was obtained from Krungthepchemi, Thailand. Hydroxypropyl methylcellulose (HPMC) capsules size 00 were obtained from Zhejiang Yuexi Capsule, Shaoxing, China. Dulbecco’s modified Eagle’s medium (DMEM), fetal bovine serum (FBS), Hank’s balanced salt solution (HBSS), phosphate-buffered saline (PBS), l-glutamine, penicillin, and streptomycin were supplied by Gibco, UK. 2,2ʹ-Azobis(2-methylpropionamidine) dihydrochloride (AAPH) and 2ʹ,7ʹ-dichlorodihydrofluorescein diacetate (DCFH-DA) were supplied by Sigma-Aldrich, MO, USA.

### Determination of apigenin solubility

To formulate the SNEDDS, the selection of suitable oils, surfactants, and cosurfactants is important to enhance the solubility of actives and the loading efficiency. The components used in SNEDDS should maximize drug solubility and have good miscibility with each other to obtain a stable formulation^15^. Therefore, the solubility of apigenin was firstly determined in the oils (Gelucire 44/14, Myritol 318, sunflower oil, and corn oil), surfactants (PEG-40 hydrogenated castor oil, Tween 20, Tween 80, Span 20, and Span 80), and cosurfactants (PEG 400 and Transcutol HP). Briefly, an excess amount of apigenin was weighed into 0.5 g of each excipient. The obtained mixtures were mixed using a vortex mixer and kept in a shaking water bath at 100 rpm and 37 ± 2 °C for 48 h. After equilibrium was achieved, the equilibrated sample was centrifuged at 8000 rpm for 20 min. The excess undissolved apigenin was discarded, and the supernatant was collected. The concentration of apigenin was analyzed by the high-performance liquid chromatography (HPLC). The experiments were performed in triplicate. The excipient in which apigenin was the most soluble was selected to form the pseudoternary phase diagram.

### Pseudoternary phase diagram

Pseudoternary phase diagrams were constructed to identify the nanoemulsions region for optimizing the concentrations of oil, surfactant, and cosurfactant and determining their mixing ratios. The oil, surfactant, and cosurfactant in which apigenin was the most soluble were chosen for the construction of a pseudoternary phase diagram using the water titration method at room temperature. Each component represented an apex of the triangle, and their total was kept at 100%. For each phase diagram, each specific ratio of surfactant:cosurfactant (S_mix_) was mixed with oil in the following weight ratios: 10:0, 9:1, 8:2, 7:3, 6:4, 5:5, 4:6, 3:7, 2:8, 1:9, and 0:10. Each weight ratio of oil and S_mix_ was titrated slowly with water. Visual observation was carried out to determine the phase clarity. The S_mix_ ratios by weight of Tween 80 and PEG 400 at 1:0, 1:1, 1:2, and 2:1 were investigated. The ratios of S_mix_ and the component ratios of the mixtures of S_mix_ with oil that created nanoemulsions (showing clear/transparent and flowable mixtures) were selected.

### Preparation of apigenin-loaded SNEDDS

Different formulations were carefully chosen from the zone of nanoemulsions from each constructed phase diagram. From the obtained phase diagrams, the ratio of S_mix_ and the component ratios of mixtures of S_mix_ with oil at the specific S_mix_ ratio that provided the largest area of nanoemulsions in the phase diagram indicated suitable ratios for the preparation of SNEDDS. The chosen formulations had a specific S_mix_ ratio of 1:1 and concentrations of Gelucire 44/14, Tween 80, and PEG 400 in the ranges of 5–40% w/w, 30–47.5% w/w, and 30–47.5% w/w, respectively, as shown in Table 1. The apigenin-free SNEDDS (blank) was prepared by mixing Gelucire 44/14 with Tween 80:PEG 400 (S_mix_) according to the formula ratio. For apigenin-loaded SNEDDS, 0.5% w/w of apigenin was added to the blank SNEDDS and continuously stirred at 40 °C for 30 min. The excess undissolved apigenin was separated to obtain a clear mixture. The apigenin-loaded SNEDDS were kept in tightly closed glass vials at room temperature for further study.

**Table 1:** Compositions and physical characterizations of all formulations of apigenin-loaded SNEDDS (GTP) and blank SNEDDS (BGTP).

Formulation	Gelucire 44/14 (%)	Tween 80 (%)	PEG 400 (%)	Apigenin (%w/w)	Z-ave (nm)	PDI	Zeta potential (mV)	%T	Appearance	Emulsification time (s)
GTP4060	40.0	30.0	30.0	0.5	58.61 ± 1.56	0.60 ± 0.05	− 9.24 ± 0.14	99.43 ± 0.22	Translucent	32
GTP3565	35.0	32.5	32.5	0.5	55.89 ± 2.34	0.59 ± 0.04	− 13.67 ± 0.32	99.43 ± 0.34	Translucent	20
GTP3070	30.0	35.0	35.0	0.5	26.86 ± 4.96	0.44 ± 0.16	− 3.49 ± 1.22	99.66 ± 0.29	Clear	16
GTP2575	25.0	37.5	37.5	0.5	11.32 ± 0.05	0.19 ± 0.03	− 4.60 ± 1.22	99.14 ± 0.62	Clear	17
GTP2080	20.0	40.0	40.0	0.5	11.00 ± 0.04	0.06 ± 0.02	− 3.35 ± 0.24	99.54 ± 0.29	Clear	14
GTP1585	15.0	42.5	42.5	0.5	10.77 ± 0.14	0.05 ± 0.00	− 1.19 ± 1.28	99.54 ± 0.22	Clear	13
GTP1090	10.0	45.0	45.0	0.5	10.59 ± 0.08	0.09 ± 0.01	− 5.22 ± 3.63	99.60 ± 0.19	Clear	6
GTP0595	5.0	47.5	47.5	0.5	10.27 ± 0.12	0.09 ± 0.05	− 0.89 ± 1.08	99.43 ± 0.11	Clear	4
BGTP4060	40.0	30.0	30.0	–	16.56 ± 0.12	0.22 ± 0.01	− 1.25 ± 0.56	99.54 ± 0.30	Clear	–
BGTP3565	35.0	32.5	32.5	–	17.08 ± 0.09	0.20 ± 0.01	0.61 ± 1.54	99.60 ± 0.22	Clear	–
BGTP3070	30.0	35.0	35.0	–	11.53 ± 0.43	0.12 ± 0.03	− 1.07 ± 1.51	99.54 ± 0.30	Clear	–
BGTP2575	25.0	37.5	37.5	–	13.76 ± 0.09	0.21 ± 0.01	− 0.69 ± 0.78	100.12 ± 0.19	Clear	–
BGTP2080	20.0	40.0	40.0	–	13.66 ± 0.03	0.21 ± 0.01	− 5.51 ± 2.50	100.00 ± 0.13	Clear	–
BGTP1585	15.0	42.5	42.5	–	10.46 ± 0.08	0.07 ± 0.02	− 0.75 ± 1.19	99.89 ± 0.19	Clear	–
BGTP1090	10.0	45.0	45.0	–	15.25 ± 0.49	0.19 ± 0.01	− 2.38 ± 1.83	99.66 ± 0.65	Clear	–
BGTP0595	5.0	47.5	47.5	–	15.51 ± 0.35	0.18 ± 0.01	− 4.12 ± 4.58	99.83 ± 0.22	Clear	–

*%T* percent transmittance.

### Determination of the droplet size, polydispersity index, and zeta potential

The mean droplet size (z-ave), polydispersity index (PDI), and zeta potential (ZP) of all formulations were measured by a laser scattering technique using a Zetasizer Nano ZS (Malvern Instruments, UK) at the temperature of 25 °C. Before the measurements, each SNEDDS sample was diluted with deionized water at a ratio of 1:100 v/v and mixed well for 1 min. Each measurement was performed in triplicate.

### Percent transmittance

The transmittance after dilution with water determines the clarity-turbidity of SNEDDS, which indicates the optical isotropy and thermodynamic stability. An isotropic nature is presented when a percent transmittance (%T) is closer to 100%, indicating the formation of globules in the nanosized range^16,17^. The optical clarity of the SNEDDS was determined by spectrophotometry using a BMG Nano (UV–Vis) microplate reader (BMG LABTECH GmbH, Germany). SNEDDS diluted with deionized water at a ratio of 1:100 v/v were measured for the %T at 500 nm with deionized water as a blank. Each sample was analyzed in quadruplicate.

### Self-emulsification time

The emulsification process and the tendency of the SNEDDS to emulsify spontaneously were assessed by measuring the self-emulsification time. A decrease in self-emulsification time indicates an increase in the efficiency of SNEDDS to form nanoemulsions. To compare the self-emulsification time of each formulation, 0.1 mL of SNEDDS was added dropwise to 10 mL of deionized water maintained at 37 ± 2 °C. Each sample was gently and continuously agitated. The time required for the complete formation of nanoemulsions was observed. All measurements were repeated in triplicate.

### Morphological characterization

The morphology of the SNEDDS was investigated by TEM (JEM-1400, JEOL, MA, USA). SNEDDS were diluted with deionized water at a ratio of 1:100 v/v and mixed to obtain nanoemulsions. One drop of diluted sample was deposited on a film-coated 200 mesh copper grid and allowed to stand for 20 min, after which any excess fluid was removed with filter paper. The grid was later stained with 1% phosphotungstic acid and dried for 15 min before examination with a transmission electron microscope with an accelerating voltage at 80 kV and × 100,000 magnification.

### Thermodynamic stability

SNEDDS should undergo solubilization to form stable nanoemulsions without precipitation, creaming, or cracking. Therefore, the thermodynamic stability must be evaluated following these topics.

#### Centrifugation

Each apigenin-loaded SNEDDS formulation was centrifuged using a Universal 230 (Hettich, Germany) at 3500 rpm for 30 min to determine its stability as an isotropic single-phase system.

#### Heating–cooling cycle

Each apigenin-loaded SNEDDS formulation was kept in a tightly closed glass container, incubated at 4 ± 2 °C for 48 h and 40 ± 2 °C for 48 h as 1 cycle. The experiment was conducted in 6 cycles.

#### Freeze–thaw cycle

Freeze–thaw testing involved freezing each apigenin-loaded SNEDDS formulation at − 20 ± 2 °C for 48 h and then allowing it to thaw at 25 ± 2 °C for 48 h, representing one complete cycle. The experiment was conducted in 3 cycles.

From the thermodynamic stability test, signs of phase separation, creaming, or cracking were noted. Any formulation showing any sign of instability was discarded.

### HPLC analysis of apigenin

The amount of apigenin was quantified by a validated reversed-phase HPLC (RP-HPLC) equipped with a UV/VIS detector (SHIMADZU Series 20AD, Japan). Apigenin was separated on a C18 column (Promosil C18, 4.5 × 250 mm, 5 µm) using methanol, acetonitrile, acetic acid, orthophosphoric acid, and water at a ratio of 40:20:0.05:0.05:40 by volume as the mobile phase at a flow rate of 0.6 mL/min with a detection wavelength of 352 nm. The column temperature was set to 30 °C. The standard curve of apigenin was linear in the concentration range of 0.05–40 µg/mL. Good linearity with a coefficient of determination (R^2^) > 0.999 was achieved. The relative standard deviation (%RSD) of intraday and interday precision of the assay method for apigenin was below 6%, and the accuracy was within the range of 95–105%. The limit of quantification and limit of detection were 0.64 and 0.21 µg/mL, respectively.

### Drug loading

Each apigenin-loaded SNEDDS formulation was assayed to determine the amount of apigenin that was loaded. The concentration of apigenin in SNEDDS sample was analyzed by HPLC as previously mentioned. A certain weight of the sample was completely dissolved in DMSO and stirred with a vortex mixer. The amount of apigenin loaded in each SNEDDS formulation was calculated as the percent drug loading as shown in Eq. (1) and was compared to the initial drug load (0.5% w/w). Each determination was repeated in triplicate.

1 $$\text{\%}\text{ Drug loading}\text{ } =  \left(\frac{\text{Amount of loaded drug}}{\text{Amount of total ingredients in the formulation}}\right)\times {100}\text{.}$$

### Encapsulation efficiency

The encapsulation efficiency was calculated to determine the amount of apigenin that could be encapsulated in the nanoemulsions formed after dilution of the preconcentrated SNEDDS with water. Each apigenin-loaded SNEDDS formulation (with a known amount of apigenin) was transferred to a small safe-lock test tube and diluted with deionized water at a ratio of 1:5 (SNEDDS:water) to obtain nanoemulsions. Each sample was then centrifuged at 14,000 rpm for 2 h at 4 °C. The supernatant was collected, and the free drug (unentrapped apigenin) was quantified by HPLC. Each determination was performed in triplicate. The encapsulation efficiency was calculated according to the following equation.

2 $$\text{\% Encapsulation efficiency = }\left(\frac{\text{Total amount of drug} - \text{Amount of free drug}}{\text{Total amount of drug} }\right)\times {100}\text{.}$$

### Fourier transform infrared (FT-IR) spectroscopy

FT-IR was employed to evaluate the possible interactions between apigenin and the other SNEDDS components. FT-IR spectra were obtained over a range of 4000–400 cm^−1^ using the FT-IR spectrometer (Nicolet iS5, Thermo Scientific, WI, USA) equipped with a diamond crystal iD7 ATR (attenuated total reflection) accessory. Each measurement represents an average of 32 scans with a resolution of 4 cm^−1^. Each SNEDDS composition, including pure apigenin, Gelucire 44/14, Tween 80, and PEG 400, the physical mixture of apigenin with other SNEDDS components, and the SNEDDS formulations GTP2575 and GTP3070, was evaluated.

### Differential scanning calorimetry (DSC)

The thermal properties of the samples were investigated using a DSC8000 (PerkinElmer, Llantrisant, UK). All samples (approximately 10 mg) were accurately weighed in a crimped aluminum pan. DSC thermograms were generated by heating at a heating rate of 10 °C/min over a temperature range of 30–400 °C under nitrogen gas flowing at 20 mL/min. Each SNEDDS composition, including pure apigenin, Gelucire 44/14, Tween 80, and PEG 400, the physical mixture of apigenin with other SNEDDS components, and the SNEDDS formulations GTP2575 and GTP3070, was evaluated.

### In vitro dissolution profile

The apigenin-loaded SNEDDS formulations GTP2575 and GTP3070 were selected for evaluating the in vitro dissolution because both formulations offered nearly 100% transmittance, an appropriate size with a narrow size distribution, a quick self-emulsification time, and great drug loading and encapsulation efficiency. In addition, the surfactant ratio above 75% did not affect the particle size and might cause GI irritation^18^. Therefore, formulations GTP2575 and GTP3070 were preferred for in vitro dissolution study.

Dissolution was performed by a dissolution apparatus type II (a paddle) (Vision G2 Elite 8, Hanson, CA, USA). The SNEDDS formulations GTP2575 and GTP3070 containing 5 mg of apigenin were added to HPMC capsules number 00. An equivalent 5 mg of apigenin coarse powder added to HPMC capsules using lactose monohydrate as the filler was the control. Each capsule was placed inside a sinker loaded with 900 mL of dissolution media (buffer solutions pH 1.2, 4.5, and 6.8) at 37.0 ± 0.5 °C with a rotating speed of 100 rpm. During the study, 5 mL of dissolution medium was removed at predetermined time intervals (10, 15, 20, 30, 45, 60, 90, and 120 min), and 5 mL of fresh buffer solution was replaced. The amount of apigenin dissolved in the dissolution media was determined by HPLC. The dissolution experiment was carried out in triplicate. The dissolution profile was plotted with the percent apigenin dissolved on the y-axis and time on the x-axis. The dissolution profiles of GTP2575, GTP3070, and the coarse powder in the dissolution media buffer pH 1.2, 4.5, and 6.8 were compared. The dissolution results of the SNEDDS formulations GTP2575 and GTP3070 in pH 1.2 buffer solution were fitted by determining the R^2^ with various kinetic models such as zero-order (a constant release rate independent of the drug remaining), first-order (an exponential decay of the drug remaining), Higuchi (matrix systems where drug release is controlled by diffusion), Hixon–Crowell (release based on the change in the cube root of the drug remaining), and Korsmeyer–Peppas (an extension of the Hixson-Crowell model that incorporates a release exponent). Korsmeyer–Peppas is known as the “power law” describing drug release following several kinetic mechanisms and is also used when the controllable process mechanism is incomprehensible, such as with a combination of more than one type of release mechanism^19,20^. The value of n (a release exponent) indicates the mechanism of drug transport. If n takes the value of 0.45 or less, the drug release mechanism follows Fickian (case I) diffusion, and a value of 0.89 or more indicates swelling-controlled drug release (case II). Values of n between 0.45 and 0.89 can be regarded as an indicator for both phenomena (anomalous transport).

### Intracellular antioxidant activity

The quantitative cellular antioxidant activity (CAA) assay is a useful method employed for determining the bioavailability of food antioxidants because it can account for several additional factors, such as cellular uptake and metabolism. The CAA assay uses a fluorescent probe (DCFH-DA) to monitor the inhibition of peroxyl radical-induced oxidation inside the cell. The ester form (DCFH-DA) is nonionic and nonpolar and can be transported across the cell membrane. Inside the cell, DCFH-DA is deacetylated by endogenous cellular esterase and left in its oxidizable DCFH form. Once the free radical generator AAPH is added to the system, peroxyl radicals begin to form. The addition of antioxidants that can enter the cell can compete with and quench the radicals, preventing DCFH from being oxidized to form DCF (fluorescent). The CAA unit takes into account the relative areas under the curves of the samples compared to the control. Caco-2 cells, intestinal barrier models, are an appropriate model for assessing the antioxidant activity, especially for the phenolic compound^21^ being used in this study.

The quantitative CAA of SNEDDS formulations GTP2575 and GTP3070 was evaluated in Caco-2 cells modified from the study of Kellett et al.^21^. Caco-2 cells were cultured in advanced DMEM supplemented with 10% endotoxin-free, heat-inactivated FBS, 1% l-glutamine, 1% penicillin (10,000 U/mL), and 1% streptomycin (10,000 μg/mL). Each test sample of SNEDDS was prepared by diluting with serum-free culture media to obtain an equivalent concentration of apigenin (14 µg/mL). The working solution (25 μM DCFH-DA in serum-free culture media) was prepared from a 12.5 mM DCFH-DA stock solution in MeOH. A 600 μM AAPH solution was prepared from a 60 mM AAPH stock solution in HBSS before experimental use. After Caco-2 cells reached confluence in a T-25 culture flask, the cells were washed twice with sterile PBS (pH 7.4) and dissociated from the surface using trypsin–EDTA. The cells were seeded in 96-well, black, flat-bottom tissue culture-treated dishes (Corning Costar, ME, USA). A density of 60,000 cells in 100 µL of culture media was added to each well and incubated until they reached confluency (24 h). The growth medium was removed after confluence was achieved, and the cells were then washed with PBS to remove any nonadherent and dead cells.

At the time of the experiment, 50 µL of 25 μM DCFH-DA working solution was added to each well, followed by 50 µL of a test sample. As a result, the final concentration of apigenin of 7 μg/mL in each well was obtained. As a control, 50 µL of DCFH-DA working solution and serum-free culture media (no sample included) were utilized. Once the DCFH-DA and test samples were added, the cells were placed in the incubator for 1 h at 37 °C. After 1 h, the cells were washed with PBS 3 times to ensure that any antioxidant effect observed later in the assay was due only to the compounds internalized by the cells. Then, 100 µL of the free radical generator, 600 µM AAPH solution, was added. The cells were then immediately placed in a BMG CLARIOstar (BMG LABTECH, NC, USA), where real-time fluorescence was measured initially and every five minutes for 1 h (a total of 13 readings were taken). The fluorescence was measured at an excitation wavelength of 485 nm and an emission wavelength of 538 nm. The antioxidant activity of test samples was quantified by evaluating the percent reduction in fluorescence. The area under the curve (AUC) was determined after generating the curve of the 13 fluorescence responses from the 1 h assay. The percent reduction, or the CAA unit, was calculated using Eq. (3). The samples were evaluated in triplicate and reported as the mean ± SD. An equivalent amount of apigenin coarse powder in 1% v/v DMSO was prepared and assayed using a similar method. A 0.01%w/v ascorbic acid solution was employed as the positive control.

3 $$\text{CAA unit = \% reduction = }\left[\text{1 }- \, \left(\frac{{\text{AUC}}_{\text{sample}}}{{\text{AUC}}_{\text{control}}}\right)\right] \, \times {100}\text{.}$$

### Statistical analysis

Statistical analysis was conducted using GraphPad PRISM version 10.0.0 (GraphPad Software, La Jolla, CA, USA). The data were analyzed by one-way ANOVA and repeated-measures ANOVA with Tukey’s comparisons to compare the mean of each sample. A value of *p* < 0.05 was considered to indicate statistical significance.

## Results

### Solubility of apigenin in various vehicles

The solubility of apigenin in various vehicles is shown in Table 2. The high solubility of the drug in the oil phase is an important factor that helps the nanoemulsions maintain the drug in its solubilized form. Among the other oils, apigenin was the most soluble in Gelucire 44/14 (7.77 ± 0.63 mg/g). Therefore, Gelucire 44/14 was chosen as an oil component in SNEDDS. The solubility of apigenin in Myritol 318, sunflower oil, and corn oil was comparable, but much lower than Gelucire 44/14. Various types of surfactants and cosurfactants were employed to evaluate the solubility of apigenin, including polyoxyethylene hydrogenated castor oil (Cremophor RH-40), polysorbates (Tween 20 and Tween 80), sorbitan esters (Span 20 and Span 80), polyethylene glycol (PEG 400), and glycol ether (Transcutol HP). The results showed that the solubilizing capacity of apigenin was dependent on the Hydrophilic-lipophilic balance (HLB) of the surfactant. Surfactants with high HLB values provided the advantage of apigenin solubilization. Apigenin was more soluble in Tween 80 (HLB 15) and Tween 20 (HLB 16.7) than Cremophor RH-40 (HLB 14–16), Span 20 (HLB 8.6), and Span 80 (HLB 4.3). Likewise, the cosurfactant PEG 400 (HLB 9.7) presented a higher capacity to solubilize apigenin than Transcutol HP (HLB 4.2). From the study, Gelucire 44/14, Tween 80, and PEG 400 showed the highest solubilization for apigenin and were chosen to prepare SNEDDS as oil, surfactant, and cosurfactant, respectively.

**Table 2:** Solubility of apigenin in various excipients (mean ± SD; n = 3).

Excipients	Solubility at 37 °C (mg/g)
Oils	
Gelucire 44/14 (Lauroyl polyoxyl-32 glycerides)	7.7686 ± 0.6332
Myritol 318 (caprylic/capric triglyceride)	0.0003 ± 0.0000
Sunflower oil	0.0004 ± 0.0000
Corn oil	0.0003 ± 0.0000
Surfactants	
PEG-40 hydrogenated castor oil	11.3645 ± 0.2659
Tween 20	20.9996 ± 1.1273
Tween 80	22.5921 ± 0.0290
Span 20	0.0013 ± 0.0000
Span 80	0.0007 ± 0.0001
Cosurfactants	
PEG 400	27.6349 ± 0.9833
Transcutol HP (diethylene glycol ethyl ether)	20.9544 ± 0.8715

### Construction of the pseudoternary phase diagram

**Figure 1:**
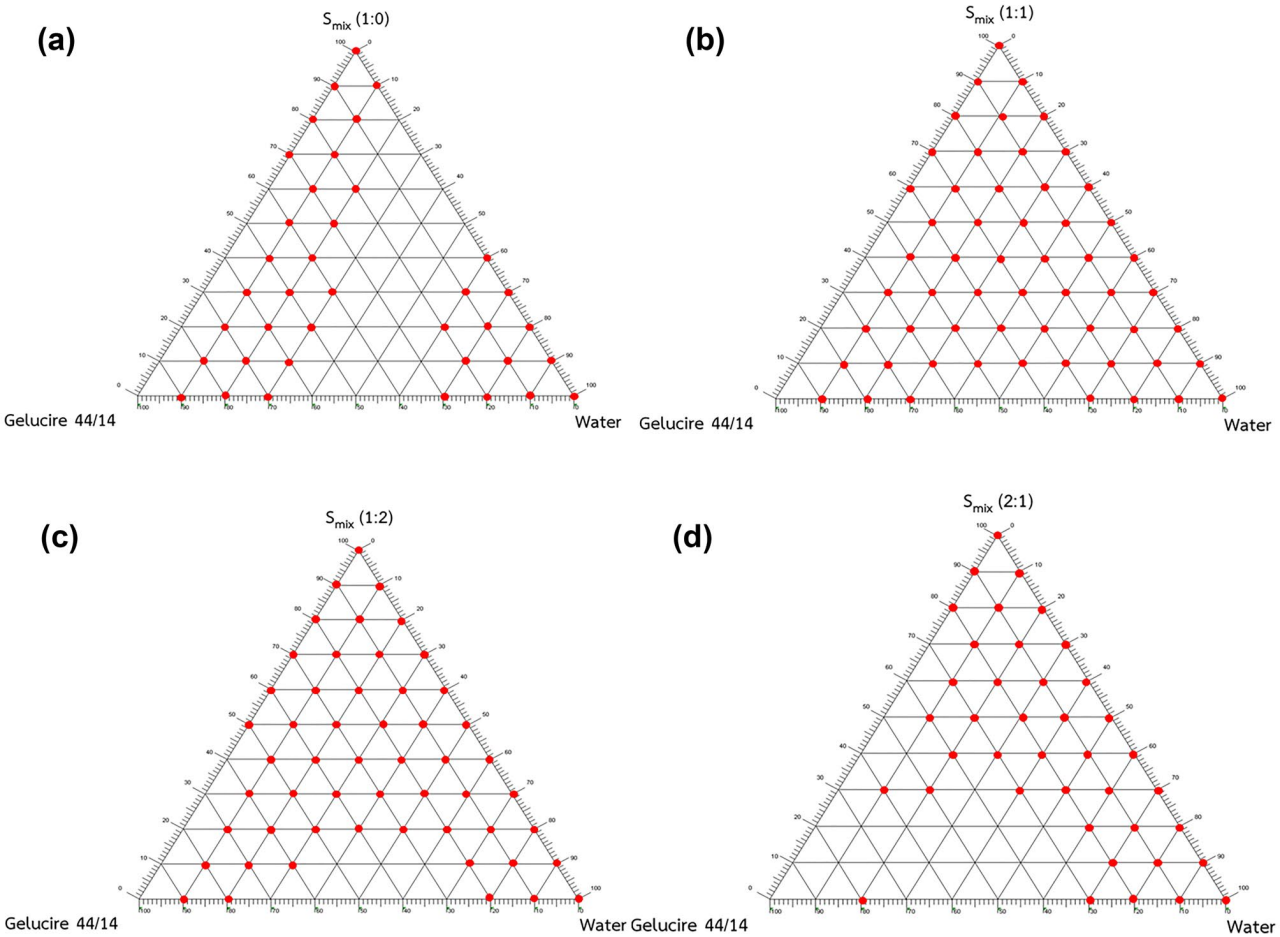
Pseudoternary phase diagram of SNEDDS. S_mix_ 1:0 (**a**); S_mix_ 1:1 (**b**); S_mix_ 1:2 (**c**); S_mix_ 2:1 (**d**). The red dots in the phase diagram present the clear/transparent nano-emulsification formation.

**Figure 2:**
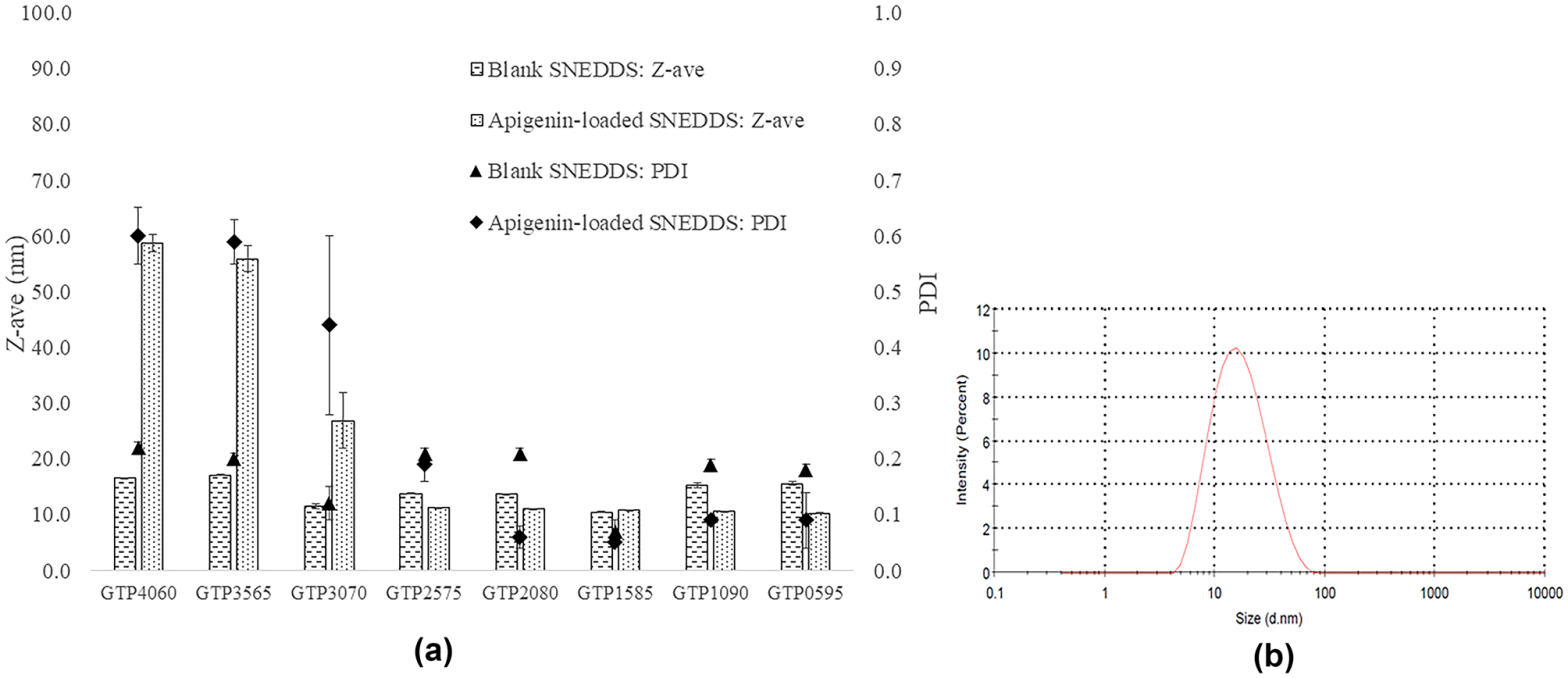
The droplet size and PDI (**a**) of blank SNEDDS and apigenin-loaded SNEDDS of all formulations, and the particle size distribution curve (**b**) of apigenin-loaded SNEDDS measured by Zetasizer Nano ZS with the average droplet size in nanoscopic range.

Phase diagrams containing Gelucire 44/14, S_mix_ (Tween 80:PEG 400), and water are shown in Fig. 1. The formation of apparent spontaneous nanoemulsions was visually observed in various ratios of S_mix_ (1:0, 1:1, 1:2, and 2:1). At S_mix_ ratios of 1:0 (Fig. 1a) and 2:1 (Fig. 1d), relatively small nanoemulsion regions were obtained with percentages of 57.58 and 60.61% of the total phase diagram area, respectively. The percent area of the nanoemulsion region was considerably increased at S_mix_ ratios of 1:2 (Fig. 1c) and 1:1 (Fig. 1b), which were 80.30 and 86.36%, respectively. Therefore, the optimal S_mix_ ratio of 1:1 was selected for the preparation of the SNEDDS because it provided the largest nanoemulsion region. In the pseudoternary phase diagram of a S_mix_ ratio of 1:1, the suitable concentrations of Gelucire 44/14, Tween 80, and PEG 400 that formed the nanoemulsions were in the ranges of 5–40% w/w, 30–47.5% w/w, and 30–47.5% w/w, respectively.

### Droplet size, polydispersity index, and zeta potential

The droplet size, PDI, and ZP of the apigenin-free and apigenin-loaded SNEDDS are shown in Table 1. As presented in Fig. 2a,b, the mean droplet size of all diluted SNEDDS formulations was in the nanometer range (< 100 nm). The particle sizes of the formulations GTP0595, GTP1090, GTP1585, GTP2080, GTP2575, and GTP3070 were comparable between their apigenin-free and apigenin-loaded forms. The PDI values of these formulations were less than or approximately 0.4, especially for the formulations with a high ratio of S_mix_ showing a PDI value of less than 0.2, indicating the uniformity of the particle size. These formulations are presented as clear solutions. However, the apigenin-loaded SNEDDS formulations GTP4060 and GTP3565 showed a translucent appearance and larger droplet size (55.89–58.61 nm) with higher PDI (> 0.5) than their blank counterparts. This result indicates that the incorporation of apigenin in the SNEDDS affected the nanoemulsions formation and might be due to an insufficient concentration of S_mix_ to solubilize apigenin. In addition, the decrease in the ratio of Gelucire 44/14 to S_mix_ brought about a decreased droplet size and PDI value of the obtained nanoemulsions presenting clear solutions. However, when the amount of Gelucire 44/14 was reduced to lower than 25% w/w (Formulations GTP2575, GTP2080, GTP1585, GTP1090, GTP0595), the droplet size or PDI value did not significantly change (*p* > 0.05). This indicated sufficient amounts of SNEDDS components to solubilize the apigenin and stabilize the nanoemulsions. The use of high concentrations of surfactants and cosurfactants may cause GI irritation. Hence, formulations with a relatively low S_mix_ ratio that was enough to provide a small particle size with narrow PDI, such as GTP2575 and GTP3070, were considered interesting.

The ZP values of the apigenin-loaded SNEDDS are shown in Table 1. The ZP values of all formulations were in the range of |− 13.67|–|− 0.89| mV, which indicates the obtained nanoemulsion droplets had a very low charge or no charge. When compared with the blank SNEDDS, apigenin-loaded SNEDDS showed a higher negative charge. This result might be due to the OH group in the apigenin molecule, which could provide negative charges to the surface of the formed nanoemulsions.

### Percent transmittance

The %T of the apigenin-loaded SNEDDS is shown in Table 1. All formulations had the %T in the range of 99.14–99.66%, implying the formation of nanoemulsions with an isotropic nature.

### Self-emulsification time

From Table 1, most formulations had a self-emulsification time of less than 30 s, implying a good ability to form nanoemulsions. Formulation GTP0595 showed the shortest self-emulsification time of 4 s. It was found that the self-emulsification time was dependent on the composition ratio of oil, surfactant, and cosurfactant. In this study, decreasing the ratio of Gelucire 44/14 and increasing S_mix_ resulted in a shorter self-emulsification time.

### Morphology

**Figure 3:**
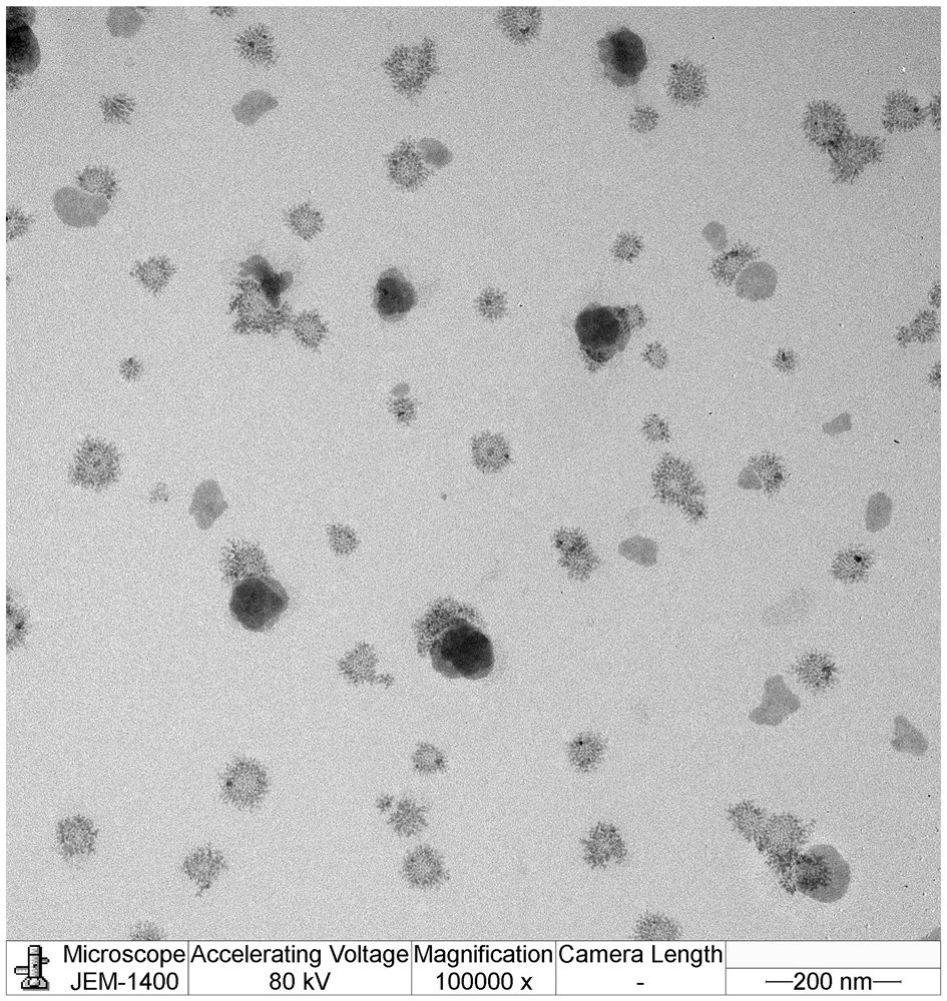
TEM images of nanoemulsions obtained from the diluted apigenin-loaded SNEDDS were in spherical shape.

A transmission electron microscopy (TEM) image of a diluted SNEDDS formulation GTP2575 is shown in Fig. 3. The morphology of the nanoemulsions from the diluted SNEDDS was spherical. The droplet size was found to be in the nanometer range, which was consistent with the results obtained from the Zetasizer Nano ZS.

### Thermodynamic stability

The thermodynamic stability results of the apigenin-loaded SNEDDS are shown in Table 3. Most of the apigenin-loaded SNEDDS were found to be thermodynamically stable after centrifugation, heating–cooling, and freeze–thaw cycles. Only formulation GTP4060 failed the freeze–thaw cycle test. After passing the freeze–thaw assessments, the formulation GTP4060 increased in turbidity, which might be due to the insufficient amounts of surfactant and cosurfactant to maintain the complete solubility of apigenin.

**Table 3:** Thermodynamic stability of all formulations of apigenin-loaded SNEDDS (observation of the signs of phase separation, creaming, or cracking).

Formulation	Observation based on thermodynamic stability test			Inference
	Centrifugation	Heating–cooling cycle	Freeze–thaw cycle	
GTP4060	–	–	x (turbid)	Fail
GTP3565	–	–	–	Pass
GTP3070	–	–	–	Pass
GTP2575	–	–	–	Pass
GTP2080	–	–	–	Pass
GTP1585	–	–	–	Pass
GTP1090	–	–	–	Pass
GTP0595	–	–	–	Pass

### Drug loading and encapsulation efficiency

The drug loading and encapsulation efficiency of all apigenin-loaded SNEDDS formulations are presented in Table 4. The drug loading of all formulations was in the range of 0.38–0.45% w/w, which was equal to 75.72–90.10% of the initial drug load (0.5% w/w). According to the preparation process, an excess amount of undissolved apigenin was separated to obtain a final clear formulation. Therefore, the different drug loadings could indicate the different capacities of each formulation to solubilize the active compound. Each formulation had a significant difference in loading capacity (*p* < 0.05), except for formulations GTP2080 and GTP1090. A larger drug loading value indicates a better ability to solubilize the active compound. The types and amounts of the SNEDDS components influenced the apigenin loading. GTP2575 and GTP3070 were the two highest-drug loading formulations, with 90.10 ± 0.24% and 88.59 ± 0.07% of the initial drug load, respectively. It might be implied that the concentration ratios of Gelucire 44/14 and S_mix_ of both formulations were the most suitable ratios for solubilizing the apigenin, leading to the highest drug loading. Additionally, the %encapsulation efficiency of all formulations varied between 80 and 85%, with the maximum value displayed by GTP2575 of 84.20 ± 0.03%, indicating the most appropriate component ratio to provide the highest entrapment efficiency.

**Table 4:** The %drug loading, %compared to the initial drug load (0.5%w/w), and %encapsulation efficiency of all formulations of apigenin-loaded SNEDDS (mean ± SD; n = 3).

Formulation	%Drug loading (%compared to initial load (0.5%w/w))	%Encapsulation efficiency
GTP4060	0.42 ± 0.00 (83.08 ± 0.20)^a^	82.64 ± 0.05^h^
GTP3565	0.42 ± 0.00 (84.53 ± 0.09)^b^	82.55 ± 0.02^i^
GTP3070	0.44 ± 0.00 (88.59 ± 0.07)^c^	82.14 ± 0.05^j^
GTP2575	0.45 ± 0.00 (90.10 ± 0.24)^d^	84.20 ± 0.03^k^
GTP2080	0.41 ± 0.00 (82.61 ± 0.05)^e^	82.20 ± 0.01^j,l^
GTP1585	0.43 ± 0.00 (85.58 ± 0.10)^f^	82.87 ± 0.03^m^
GTP1090	0.41 ± 0.00 (82.43 ± 0.10)^e^	82.26 ± 0.04^l^
GTP0595	0.38 ± 0.00 (75.72 ± 0.31)^g^	80.00 ± 0.08^n^

The Superscript letters a, b, c, d,…, n in the same column indicate a statistically significant difference between each other at a significance level of 0.05.

### FT-IR analysis

**Figure 4:**
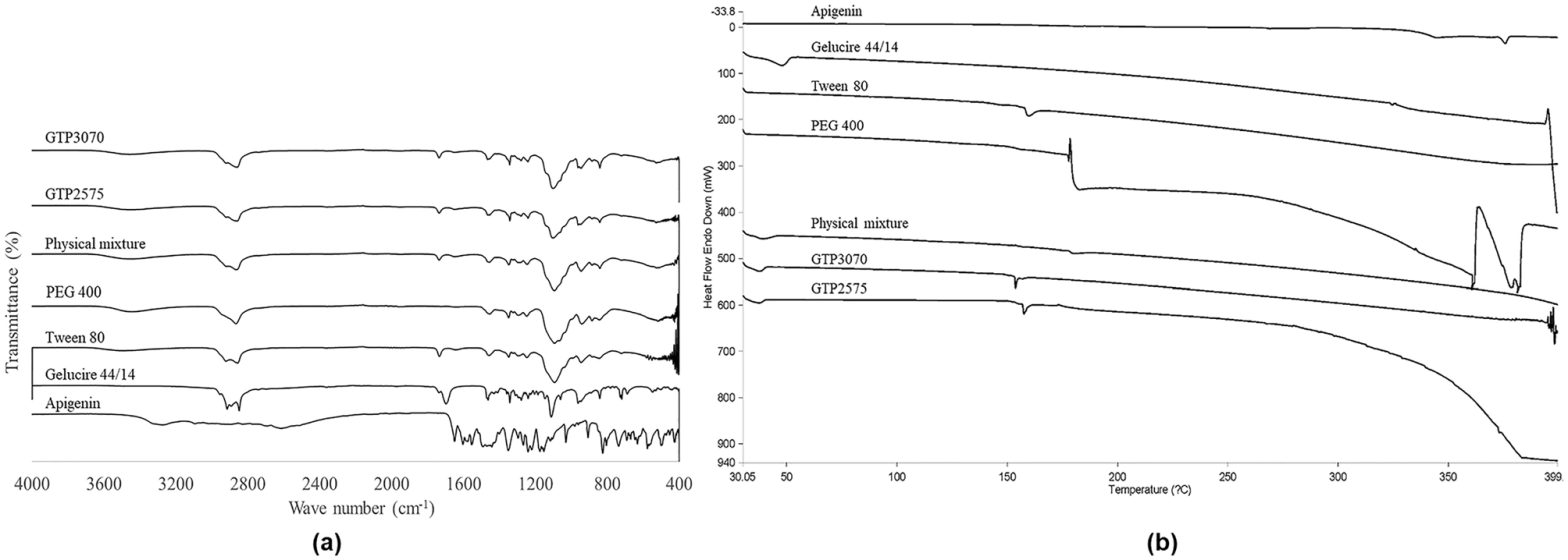
FT-IR spectra (**a**) and DSC thermograms (**b**) of apigenin, gelucire 44/14, Tween 80, and PEG 400, physical mixture, and SNEDDS formulations GTP2575 and GTP3070.

FT-IR was used to investigate the interaction between the SNEDDS components and the drug. The FT-IR spectra of apigenin and the other components of the SNEDDS, such as Gelucire 44/14, Tween 80, and PEG 400, the physical mixture of apigenin with other components of SNEDDS, and SNEDDS formulations GTP2575 and GTP3070 are presented in Fig. 4a. The apigenin spectrum exhibited characteristic peaks at 3279 cm^−1^, corresponding to O–H bonding; 2616 cm^−1^, corresponding to C–H bending; 1650 and 1605 cm^−1^, representing C=O stretching; and 1352 and 1175 cm^−1^, representing C–C stretching. The obtained FT-IR data were in line with the data reported by Aldawsari et al.^22^ The FT-IR spectrum of Gelucire 44/14 showed characteristic peaks at 2915 and 2848 cm^−1^ (C–H stretching), 1698 cm^−1^ (C=O stretching), and 1112 cm^−1^ (C–O stretching). The FT-IR spectrum of Tween 80 displayed a broad peak centered at 3510 cm^−1^, which was assigned to O–H stretching. The absorption bands at 2922 and 2856 cm^−1^ (C–H stretching), 1735 cm^−1^ (C=O stretching), and 1093 cm^−1^ (C–O–C stretching) were observed. In the case of PEG 400, typical bands of these polymers can be observed at wavenumbers of 3438 cm^−1^ (O–H stretching), 2866 cm^−1^ (–CH_3_ stretching), and 1094 cm^−1^ (C–O–C stretching). The presence of the characteristic peaks of the drug and carriers (SNEDDS components) with no additional peaks in the physical mixture and the SNEDDS formulations negated that there was any interaction between the drug and carriers, suggesting that apigenin was stable in the systems.

### DSC analysis

To assess the influence of apigenin incorporation on the physical state and thermal behavior of the SNEDDS, the DSC thermograms of pure apigenin, Gelucire 44/14, Tween 80, PEG 400, the physical mixture of apigenin with other SNEDDS components, and the SNEDDS formulations GTP2575 and GTP3070 are shown in Fig. 4b. The apigenin thermograms revealed a sharp melting peak at 375.8 °C, indicating its crystalline nature^23^. The DSC thermogram of Gelucire 44/14 presented an endothermic peak at 47.9 °C. Tween 80 showed an endothermic peak at approximately 150 °C, corresponding to its flash point^24,25^. PEG 400 showed a change in the baseline at a temperature of approximately 180 °C and started to decompose at a temperature higher than 350 °C. The physical mixture and the SNEDDS formulations GTP2575 and GTP3070 showed two endothermic peaks at approximately 40 and 150 °C, corresponding to Gelucire 44/14 and Tween 80, respectively. The decrease in the melting point of Gelucire 44/14 in the physical mixture and SNEDDS thermograms might be due to the solvent effect of Tween 80. There was no apigenin melting peak in the physical mixture or SNEDDS, which indicated that apigenin could completely dissolve and be uniformly distributed in the molten carrier of the SNEDDS formulation^26,27^. In accordance with the FT-IR results, no new peaks were noticed in the DSC thermograms of the physical mixture or SNEDDS, negating the possibility of drug-carrier interactions.

### In vitro dissolution

**Figure 5:**
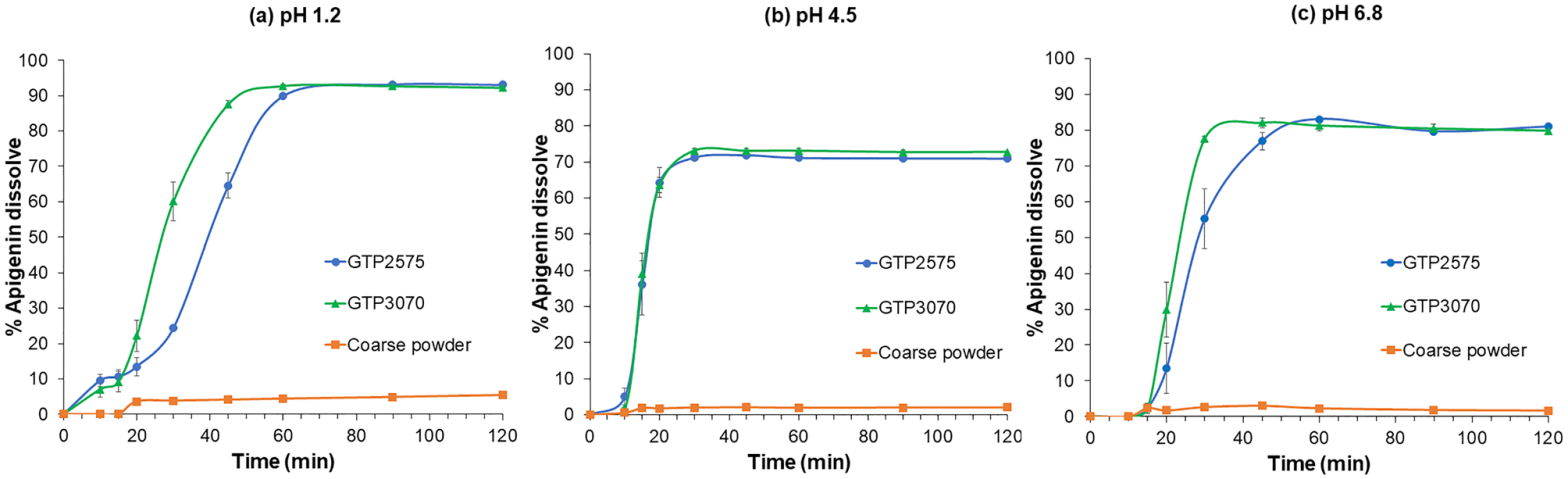
Dissolution profiles of apigenin-loaded SNEDDS formulations GTP2575, GTP3070, and coarse powder in the dissolution media buffer pH 1.2 (**a**), 4.5 (**b**), and 6.8 (**c**).

The dissolution profiles of GTP2575, GTP3070, and the coarse powder in the dissolution media buffer pH 1.2, 4.5, and 6.8 are shown in Fig. 5a–c, respectively. The percent apigenin that had dissolved at 120 min from GTP2575 and GTP3070 was approximately 90%, 70%, and 80% in dissolution media at pH 1.2, 4.5, and 6.8, respectively. In contrast, the percentage of apigenin coarse powder dissolved was only 5%, 2%, and 2%, respectively. It might be concluded that the %dissolved apigenin from SNEDDS formulations GTP2575 and GTP3070 was significantly higher than that from apigenin coarse powder (*p* < 0.05). When comparing the dissolution profiles of GTP2575 and GTP3070 in pH 4.5 buffer, there was no significant difference between them (*p* > 0.05). In addition, it was observed that GTP3070 had a faster initial dissolution rate (15–45 min) than GTP2575 in dissolution media at pH 1.2 and 6.8. However, both apigenin-loaded SNEDDS formulations exhibited a comparable percentage of apigenin dissolved after 120 min (*p* > 0.05).

Table 5 depicts the apigenin release kinetics from the SNEDDS. The release pattern of apigenin fitted best to the Korsmeyer-Peppas model (R^2^ = 0.9647 and 0.9308, and n value = 0.7829 and 0.6106 for GTP2575 and GTP3070, respectively).

**Table 5:** The coefficient of determination (R^2^) and kinetics of apigenin release from SNEDDS formulations GTP2575 and GTP3070 in pH 1.2 buffer solution.

Formulation	Zero-order	First-order	Higuchi	Hixon–Crowell	Krosmeyer–Peppas	
	(R^2^)	(R^2^)	(R^2^)	(R^2^)	(R^2^)	(n)
GTP2575	0.8438	0.8865	0.8510	0.8767	0.9647	0.7829
GTP3070	0.7215	0.7881	0.8156	0.7700	0.9308	0.6106

### Intracellular antioxidant activity

The antioxidant activity of GTP2575 and GTP3070 is presented as CAA units in Table 6. The CAA units of GTP2575 and GTP3070 were 52.25 ± 5.55% and 54.64 ± 7.19%, respectively, in comparison to the nonantioxidant-treated control. In addition, the positive control (0.01% w/v ascorbic acid) and the equivalent amount of apigenin coarse powder in 1% v/v DMSO showed a CAA unit of 21.61 ± 7.41% and 12.70 ± 5.14%, respectively. The higher CAA units of the apigenin-loaded SNEDDS indicated a decrease in fluorescence, meaning that the apigenin-loaded SNEDDS showed higher antioxidant potential when compared to the apigenin coarse powder.

**Table 6:** Cellular antioxidant activity (CAA) of apigenin-loaded SNEDDS, the coarse powder of apigenin suspended in the cell culture media (DMEM), and the standard antioxidant (0.01%w/v ascorbic acid) compared with the non-antioxidant treated control (mean ± SD; n = 3).

Test sample	AUC^#^	CAA unit (%reduction)
Non-antioxidant treated control	11,412,424 ± 1,257,737	–
GTP2575	5,412,300 ± 373,722	52.25 ± 5.55*,**
GTP3070	5,165,620 ± 857,486	54.64 ± 7.19*,**
Apigenin coarse powder in 1%v/v DMSO	9,951,772 ± 1,061,408	12.70 ± 5.14*
0.01%w/v ascorbic acid	8,959,896 ± 1,441,639	21.61 ± 7.41*

^#^AUC is the areas under the DCF fluorescence response curve over times.

*The CAA unit of the sample was significantly different from the non-antioxidant treated control, at *p* < 0.05.

**The CAA unit of the sample was significantly different from the apigenin coarse powder in 1%v/v DMSO, at *p* < 0.05.

## Discussion

From the study, the best formulations of apigenin-loaded SNEDDS were GTP2575 and GTP3070 showing good physicochemical properties and stability. Gelucire 44/14 was considered as the oil component that gave the highest solubilization for apigenin over the medium-chain triglycerides (Myritol 318) and vegetable oils (sunflower oil and corn oil). Gelucire 44/14 molecules are composed of 20% mono-, di-, and triglycerides, 72% mono- and diunsaturated fatty acid esters of PEG 1500, and 8% free PEG 1500^28^. According to this unique structure, which consists of different moieties in the molecule, including surfactants (mono- and diesters of PEG), cosurfactants (monoglycerides), and an oily phase (di- and triglycerides), it is able to increase the solubilization of poorly water-soluble drugs and their bioavailability^29^. The HLB value of surfactants and cosurfactants in SNEDDS also affected the solubilization of apigenin. To obtain a stable formulation with a small and narrow size distribution, the HLB value of a surfactant and cosurfactant should be as close as that required by the oil, which leads to easier achievement of nanoemulsions after diluting with water^30^.

The results from in vitro dissolution study indicated the potential of SNEDDS to solve the limitation of apigenin by improving the dissolution. For this reason, the oral absorption and bioavailability of apigenin administered as SNEDDS might be enhanced. It was observed that in buffer pH 1.2 and 6.8, GTP3070 had a faster dissolution rate at the initial stage, but the percentage of apigenin dissolved was comparable at the end of the experiment compared to GTP2575. This might be due to the increased ratio of Gelucire 44/14 in GTP3070. According to the structure of Gelucire 44/14, PEG in Gelucire 44/14 is highly soluble in water. The PEG esters and monoglycerides are amphiphilic, whereas di- and triglycerides provide hydrophobic properties. When Gelucire 44/14 is in contact with water, it forms self-assembled molecules, which can enhance the solubility of apigenin in water. Therefore, an increased ratio of Gelucire 44/14 led to a faster dissolution rate. The dissolution profile of apigenin-loaded SNEDDS was fitted to the Korsmeyer–Peppas model with n values between 0.45 and 0.89, indicating that the release of apigenin might be controlled by more than one process: a combination of Fickian diffusion and polymer matrix relaxation. It is possible that the solvent diffused into the capsule shell’s interior and induced capsule shell relaxation. As soon as the capsule shell was broken, the SNEDDS in the capsule solubilized and nanoemulsions were immediately formed after coming in contact with water, and then apigenin was released. Similar release anomalous transport kinetics were found in the dissolution study of zaleplon SNEDDS-loaded hard gelatin capsules by Khalifa et al.^31^. The increased dissolution of apigenin in SNEDDS affected the antioxidant potential. The SNEDDS could facilitate the permeation of apigenin into the cells both by increased dissolution providing a readily absorbable form and by the ingredient compositions in SNEDDS such as Gelucire 44/14 showing the P-gp inhibitory activity that can reduce drug efflux and enhance absorption^32^. Therefore, the antioxidant activity of apigenin administered as SNEDDS was boosted.

## Conclusions

In the present study, apigenin-loaded SNEDDS were successfully prepared and significantly enhanced the dissolution of apigenin. The optimized formulations were composed of Gelucire 44/14, Tween 80, and PEG 400, providing the highest capability of apigenin loading and showing good thermodynamic stability. They presented the increased rate and extent of apigenin dissolution compared to the intact coarse powder, confirming the efficiency of these SNEDDS to improve the dissolution, which may lead to an enhancement in oral bioavailability. The CAA of apigenin-loaded SNEDDS was approximately 50% compared to the non-antioxidant-treated control, which was also higher than the apigenin coarse powder. This implies the efficiency of the SNEDDS to increase the cellular permeation of apigenin, leading to increased antioxidant potential in the cells. The improved dissolution and antioxidant activity of apigenin-loaded SNEDDS suggest their competence to develop as novel oral supplements.

## Data Availability

The datasets generated and analyzed during the current study are available from the corresponding author upon reasonable request.
